# Nanoimaging for Molecular Pharmaceutics of Alzheimer’s and other Neurodegenerative Disorders

**DOI:** 10.4172/jmpopr.1000e107

**Published:** 2013-07-04

**Authors:** Yuri L Lyubchenko

**Affiliations:** Department of Pharmaceutical Sciences College of Pharmacy, COP 1012 University of Nebraska Medical Center 986025 Nebraska Medical Center Omaha, NE, USA

## Editorial

Misfolding and aggregation of proteins is a common thread linking a number of important human health problems. Particularly, recent studies highlighted increasing recognition of the public health importance of protein deposition diseases, including neurodegenerative disorders such as Parkinson’s disease (PD), Alzheimer’s (AD), Huntington’s and prion diseases and many other disorders [1]. Little progress has been made in the treatment of these diseases, due to a fundamental lack of knowledge of the protein self-assembly process. In fact, no effective therapeutic agents exist for Alzheimer’s disease, the most common neurodegenerative disease of aging. Understanding the mechanisms underlying self-assembly into nano-aggregates would facilitate the development of efficient therapeutic and diagnostic tools for these devastating diseases.

The first and perhaps most important elements in most neurodegenerative processes are misfolded and aggregated proteins. These are inducers of cellular stress and activators of immunity in neurodegenerative diseases and affect neuronal dysfunction and loss. All together, the accumulation of abnormal protein aggregates exert toxicity by disrupting intracellular transport, overwhelming protein degradation pathways, and/or disturbing vital cell functions. In addition, the formation of inclusion bodies is known to represent a major problem in the recombinant production of therapeutic proteins [2]. Formulation of these therapeutic proteins into delivery systems and their *in vivo* delivery are often complicated by protein association [3]. Finally, since protein refolding is frequently accompanied by transient association of partially folded intermediates, the propensity to aggregate is considered a general characteristic of the majority of partially folded proteins [4–6]. Thus, protein folding abnormalities and subsequent events underlie a multitude of pathologies and difficulties with protein therapeutic applications. Current demographic trends indicate that need for age-related and other degenerative disorders and macromolecule therapeutics will be at the forefront of future medical developments. The field of pharmaceutics therefore can be dramatically advanced by establishing a fundamental understanding of key factors leading to misfolding and self-aggregation of the various protein folding pathologies.

Despite the crucial importance of protein misfolding and abnormal interactions, very little is currently known about the molecular mechanism underlying these processes. Factors that lead to protein misfolding and aggregation *in vitro* are poorly understood, not to mention the complexities involved in the formation of protein nanoparticles with different morphologies, e.g. spherical oligomers and nanopores. Although it is well known that the same protein under pathological conditions can lead to the formation of fibrillar, pore-like, spherical, or amorphous aggregates with diverse biological consequences, the conditions leading to misfolding and the formation of such abnormal complexes are unclear, let alone their prevention. The mechanisms underlying aggregation *in vivo* in biological systems are even less clearly understood due to the difficulties in monitoring aggregates in their native state. A better understanding of the molecular mechanisms of misfolding and aggregation will facilitate rational approaches to prevent protein misfolding leading to aggregation. Also, such knowledge in conjunction with molecular modeling will fundamentally advance our understanding of cellular nanomachines and their function during protein deposition disorders. To realize this new knowledge and the associated therapeutic gains, novel experimental approaches are needed.

High-resolution methods such as x-ray crystallography, NMR, electron microscopy, and AFM have provided some information regarding the secondary structure of aggregated proteins and morphologies of self-assembled aggregates but several questions such as why the misfolded conformation of the protein is formed, and how the misfolded protein aggregates, remain unanswered. Importantly, we need to identify the conformation of misfolded protein prior to aggregation. Very likely, there are a number of such non-canonical protein conformations that exist transiently and which transform from one into another. Addressing these problems requires the use of unconventional approaches, and the development of techniques capable of probing transient conformations of proteins. The tools currently available to researchers and clinicians only provide a smeared-out picture of a living system. Because ensemble techniques average over a large population of molecules or freeze complex systems in time, much of the subtle or short-lived information is lost. The real mechanisms of life, and of disease, are played out on a very intimate and transient level through highly dynamic molecular-scale interactions. An apparently simple process, as examined by existing techniques, may actually have many steps and a variety of important intermediate states - each with their own unique dynamics. Intermediate states are stabilized by weak interactions that are typically transient and are elusive to measure. However, they are quite important as control mechanisms in several pathways. A poor understanding of the protein misfolding phenomenon is due to the paucity of experimental data describing misfolded conformations per se. Misfolded proteins aggregate readily, so structural studies have focused on relatively stable states of aggregated proteins. The conformation of the protein within aggregates may be quite different from the misfolded protein conformation prior to aggregation. The morphologies of aggregates formed under different conditions vary and it is reasonable to hypothesize that different misfolded conformation give rise to different aggregate morphologies, similar to the prion “strains” which also produce disease with different characteristics. Importantly, some of the aggregates are formed transiently, complicating structural analysis of the proteins within aggregates. Thus, it is vitally important to have ultrasensitive methods capable of probing transient protein conformations and weak intermolecular interactions on short time scales. Tantalizing experiments over the last few years have demonstrated that single molecule techniques can image intermediates, follow molecular scale events in real time, and measure a wide range of intermolecular interactions.

The very recent progress with the use of single molecule probing technique shed a new light on our understanding molecular mechanisms of the protein misfolding and aggregation process [7]. The key of the probing approach is that the proteins are anchored separately to the surface or sequestered in space in order to prevent the interaction between the protein molecules while the conditions facilitating the formation of misfolded conformations are approached. In this configuration the conformational changes are occurring within individual protein molecules without the interaction with other proteins. The major discovery of these studies is that the interaction of proteins dramatically changes their conformation [7–14]. The dimers of the proteins, compared to monomers, are stable by orders of magnitude. Therefore the dimerization of misfolded proteins is a mechanism by which the transient misfolded aggregation-prone state of a protein is stabilized. It is hypothesized that the formation of dimers is the key step for aggregation and that the long lifespans of dimeric complexes promote the selection of the misfolding-aggregation paths from non-pathologic pathways. In addition to studies of amyloid beta (Aβ) protein [9,14,15], the key factor of Alzheimer’s disease, similar mechanism was found for α-synuclein aggregation of which leads to the Parkinson’s disease development [8]. These studies suggest that the interaction between the proteins drives the formation of misfolded states, thereby stabilizing the misfolded dimers.

Advanced computational analysis and specifically molecular dynamics (MD) simulation is another tool widely used for the study of protein aggregation phenomenon [16,17]. The recent studies provided atomic resolution mechanistic model of fibril extension [18]. This model posited that after binding (docking) to the structured oligomer, the Aβ monomer conformation changes into the form present in the structured assembly. Although this study provides the explanation of how the oligomer grows but does not answer the question of how the initial oligomer structure forms. The recent MD study provided the answer [19]. They demonstrated that when two monomers approach, their structure changes dramatically and the conformational space for the two monomers becomes restricted. Therefore, misfolding of the Aβ peptide proceeds via the loss of conformational flexibility of monomers and the formation of stable dimers, and suggests their key role in the subsequent Aβ aggregation process.

In summary, the recent application of single molecule approach led to enormous progress in our understanding of molecular mechanisms of protein misfolding diseases and open prospects for future fundamental understanding of protein misfolding phenomena and building a predictive model for the self-assembly of amyloid aggregates. These studies will provide the molecular biological level basis for elucidating pathways of protein misfolding and aggregation within cells, identifying pathways or corresponding molecular mechanisms and will reveal opportunities for intervention through novel types of nanomedicine approaches. Protein misfolding is the first step in a long chain of events in development of protein misfolding diseases, so early detection of aberrant pathways and shutting down or repairing these pathologic pathways will allow us to develop novel prevention means for the diseases and eventually cure them.
